# Measuring Psychological Capital: Construction and Validation of the Compound PsyCap Scale (CPC-12)

**DOI:** 10.1371/journal.pone.0152892

**Published:** 2016-04-01

**Authors:** Timo Lorenz, Clemens Beer, Jan Pütz, Kathrin Heinitz

**Affiliations:** Department of Work and Organizational Psychology, Freie Universität Berlin, Berlin, Germany; IRCCS Istituto Auxologico Italiano, ITALY

## Abstract

With the Psychological Capital Questionnaire (PCQ) being the standard measure to assess psychological capital (PsyCap) in the context of organizations, this paper aims to broaden this domain-specific approach by introducing a measure with universal claim. Two studies were conducted to create and validate a German self-report scale (CPC-12) measuring PsyCap. We performed confirmatory factor analyses and correlations with other positive psychological constructs on the data of two German samples (N_1_ = 321; N_2_ = 202). The twelve-item CPC-12 exhibits the anticipated factorial structure with a very good model fit and associations to other constructs concur with previous findings with other measures of PsyCap.

## Introduction

Psychological capital (PsyCap) draws from the significant body of research that Seligman and Csikszentmihalyi [1] have initiated in the wake of the positive psychology movement. In shifting the focus of psychological research from human deficits like mental illnesses to human assets, strengths were scientifically studied and have been found to allow individuals, groups or even organizations to thrive and prosper [1]. Extending these findings to the work place, Luthans [2] identified psychological constructs (i.e. self-efficacy, hope, optimism, happiness, and resilience), which met the criteria of being positive, based on theory and research, and state-like open to development, change and management for performance improvement. These five constructs were labeled positive organizational behavior (POB) [2]. Luthans and Youssef [3] bundled four of these states (hope, optimism, resilience, and self-efficacy) into the higher-order construct “positive psychological capital (PsyCap)”. This construct was developed to provide practitioners with a new framework to ensure a sustainable competitive advantage via human resources [3].

PsyCap as a whole is defined as “a core psychological factor of positivity in general, and [positive organizational behavior] criteria meeting states in particular, that go beyond human and social capital to gain a competitive advantage through investment/development of ‘who you are’”[4], its parts as follows: (a) Hope refers to an individual’s motivation to succeed at a specific task in a set context and the way or means by which that task may be accomplished [5]. (b) Optimism refers to an individual’s expectancy of positive outcomes [6]. (c) Resilience refers to the ability of an individual to bounce back from adversity, uncertainty, risk or failure, and adapt to changing and stressful life demands [7, 8]. (d) Self-efficacy refers to an individual’s confidence in their ability to mobilize their motivation, cognitive resources and courses of action to achieve high levels of performance [9].

PsyCap can be distinguished from other forms of people-related capital, specifically human (an individual’s stock of knowledge, skills and abilities that can be increased by experience and/or investment in education and training) [10] and social capital (the aggregate of the actual or potential resources that are connected to the possession of a durable network of relationships) [3, 11, 12]. It influences a variety of outcomes at the individual level of particular importance for organizations and even beyond the work place [13]. Previous research however focused heavily on a domain-specific measure settled in the context of work. PsyCap is shown to be associated with desirable employee attitudes, such as staying intentions [13], job satisfaction and commitment [14, 15]. Employees high in PsyCap are found to be more empowered, which subsequently leads to less turnover intentions [16], and the reduction of absenteeism [17]. Furthermore, individuals high in PsyCap perform better than those low in PsyCap since they can draw upon more resources to pursue goals [18, 19]. Most importantly, PsyCap is shown to be developable through training interventions [15], which makes it a useful and tangible construct actually able to influence individuals and even whole organizations in a positive way.

Besides these work place-specific benefits, studies found evidence linking PsyCap to an improved psychological and physical well-being by the reduction of stress [20]. Because of the reciprocal relationship between job satisfaction and life satisfaction [21], PsyCap enhances the latter. Due to the fact that PsyCap consists of more general constructs (hope, optimism, self-efficacy and resilience) the question at hand is—Is PsyCap merely a domain-specific construct with effects solely in work-related areas or is it a much broader construct influencing many possible areas of life?

## Measuring Psychological Capital

The Psychological Capital Questionnaire (PCQ) [3] is widely recognized as the standard scale measuring PsyCap [22]. It was developed as a compound measure consisting of (modified) items from published scales for hope [23], optimism [24], resilience [25], and self-efficacy [26]. Predominantly, the PCQ was used in employee, manager and student samples [22], and its items are closely tied to the work place (i.e. “I feel confident contributing to discussions about the company's strategy.”). One can thus state that the PCQ is a domain-specific measure.

Since PsyCap is shown to be linked to outcomes of general importance for individuals [20], this study aims to design and validate a universal measure for the construct. Such a non-domain-specific measure could expedite research on PsyCap for constructs in other domains, i.e. sports and education. Therefore, we conducted two different studies. Study 1 drew from the item pool of published and proven measures for the four different PsyCap components to create a compound measure, which is deployable in a wide range of applications (including the work place). For testing convergent and discriminant validity, we additionally surveyed the existing PCQ and a measure for occupational self-efficacy. We hypothesized a strong positive association between the PCQ and our newly created measure (Compound Psychological Capital Scale– 12; CPC-12), a higher positive correlation between general self-efficacy and the CPC-12 compared to the PCQ and a lower positive one between occupational self-efficacy and the CPC-12 compared to the PCQ due to the domain specific traits of the PCQ. In study 2, we re-tested the factorial structure of the CPC-12. Furthermore, to test the external validity of the CPC-12, we also selected several important positive psychological constructs—affect, job satisfaction, satisfaction with life, subjective well-being, perceived social support, meaning of work, engagement, gratitude and personality. The specific hypotheses regarding the associations between the CPC-12 and these constructs are discussed below.

### Positive affect (PA) and negative affect (NA)

The relationship between PA and PsyCap becomes obvious upon reviewing the literature relating PA to three major components of PsyCap: resiliency, self-efficacy and optimism. These studies found that people high in PA show more effective problem resolving skills, more mature coping efforts, experience less conflict at the office [27] and furthermore that positive emotions enhance resilience in the face of adversity [28]. People who show more PA are also more optimistic and more likely to maintain a positive outlook during times of adversity [27]. Additionally, they are found to be high in personal competence and self-esteem and report higher self-efficacy [27, 29, 30]. Little, Gooty [31] also reported significant positive correlations between all four components of PsyCap and PA (*r* = .28−.68).

There is evidently a remarkable overlap between PA and PsyCap. One study even found that the predictive power of PsyCap on work performance, motivation and job satisfaction becomes insignificant once one accounts for PA [31]. Nonetheless the same study also pointed out that none of the PA-items loaded with the PsyCap constructs, meaning that besides the overlap, they are still clearly distinct constructs. For these reasons we expect a strong correlation between CPC-12 and PA. Lyubomirsky, King [27] state that NA and PA “regularly show moderate inverse relations across individuals, justifying the use of such negative states as the inverse of PA” (p. 822), thus we expect a moderate or high negative correlation between NA and PsyCap. This makes sense if you bear in mind the negative effects that PsyCap has on states like stress and anxiety [20].

### Job satisfaction

There is a clear relationship between job satisfaction and PsyCap. Studies indicate that people high in PsyCap also report higher job satisfaction [12, 13, 19]. Luthans, Avolio [19] report a positive correlation of *r* = .39, whereas the meta-analysis of Avey, Reichard [13] reports an even higher correlation of *r =* .50−.57. One explanation for this relationship is given by Avey, Reichard [13] who state: “Given the general expectancy of success derived from optimism and the belief in personal abilities derived from efficacy, those high in PsyCap report being more satisfied with their job.” (p. 132). In addition Luthans, Avolio [19] declare that employees who are hopeful and efficacious are more satisfied with their jobs due to better performance. They are confident to persist, accept challenges and put effort into achieving their goals (efficacy). Furthermore they identify subgoals and pathways to achieve them and are able to foresee and overcome obstacles by pursuing a variety of pathways (hope). We therefore expect the CPC-12 to positively correlate with job satisfaction in about the same range as stated above.

### Satisfaction with life

Previous studies on PsyCap concentrated on work-related outcomes, i.e. job satisfaction [12]. Nonetheless work and non-work life influence each other [32] and there is a positive correlation between job satisfaction and life satisfaction [33]. Ford, Heinen [32] name time-based pressure as one major reason for this mutual interaction. If you have to work extra hours in the office you will have less time to enjoy time with family or friends, which can leave you unsatisfied and vice versa. Newman, Ucbasaran [12] also stated in their meta-analysis that besides predicting higher levels of work-family conflict, low PsyCap predicts less meaning of life, things that should result in a decrease in life satisfaction. Lastly it has been reported that life satisfaction is positively related to optimism and self-esteem [34], posing another connection to PsyCap. For these reasons, we expect a moderate to high positive correlation between the CPC-12 and life satisfaction.

### Subjective well-being

Since well-being is measured in many studies as a compound construct consisting of positive and negative affect, life satisfaction and job satisfaction [35, 36], PsyCap, as expected, positively relates to well-being [22, 37–39]. Avey, Luthans [38] furthermore show that PsyCap can lead to psychological well-being over time. We therefore expect a high positive correlation between subjective well-being and the CPC-12.

### Perceived Social Support

In their study “Very Happy People” Diener and Seligman stated that satisfying social relationships are central to human happiness, so much so that happiness cannot occur without them [40]. Findings from Karademas [41] support those claims as he reports a direct positive relationship between social support and life satisfaction as well as an indirect one through optimism. Optimistic people seem to positively appraise future events. To maintain such beliefs optimists rely on a “positive evaluation of the social context and its ‘ability’ to provide the necessary support” [41]. Social support has furthermore not only been found to increase optimism [41, 42] but also to be associated with self-esteem [42]. Positive correlations between social support and optimism have been found to be moderate (*r* = .29−.41), similar to the moderate positive ones with self-esteem (*r* = .44) [41, 42]. High levels of social support have also been found to reduce mortality and to result in positive health outcomes, due to social support functioning as an “exceptionally important stress resilience factor” [43]. We expect a small to moderate positive correlation between perceived social support and the CPC-12 situated at the lower edge of the range stated above.

### Meaning of Work

When looking at meaning, its importance not only in the field of work, but for life in general [44], becomes clear. It is not just a positive influence on organizational performance or employee engagement [45]. Having meaning in life is also of great importance for living a “full life” [44], which is very desirable for many. Again there is a positive relationship with PsyCap as Coutu [46] reported a strong relationship between meaning-making and resilience. She stated that one of the things distinguishing resilient people from less resilient people is their ability to create significance and meaning in their hardships and their lives in general. In addition, she reported the effective use of constructing meaning in resilience trainings for business people. Resilient people are more likely to see themselves not as victims in adversity, but rather to recognize the lesson and learning opportunity in their hardship. They are therefore able to create meaning in difficult situations in life and at work [46]. The fact that a sense of self-worth and efficacy are found to be two important pillars in the search for meaning [47], and the finding that lower levels of PsyCap seem to result in lower levels of meaning of life [12] underline the positive relationship between meaning of work and PsyCap. We expect a moderate positive correlation between the CPC-12 and meaning of work.

### Engagement

Engagement is characterized by vigor, dedication and absorption [48]. Vigor is a very similar construct to resilience as Bakker, Schaufeli [48] describe vigorous people as mentally resilient, willing to put great effort into their work and to show persistence in the face of adversity. Resilience is not the only personal resource that has been found to reciprocally influence work engagement [48, 49]. Self-efficacy as well as optimism have been found to be equally connected to engagement [50, 51]. These personal resources show moderate positive correlations with work engagement (*r* = .29−.54) [50, 51]. Xanthopoulou, Bakker [51] explain the relationship by suggesting that people high in self-efficacy, optimism and resilience are “confident about their capabilities and optimistic about their future, and thus may identify or even create more aspects of their environment that facilitate goal attainment. This capability leads to goal confrontation and consequently to work engagement” (p. 137). It also has been explicitly stated that people high in PsyCap are more committed to their jobs [13] as the organizations they work for fulfill their needs for accomplishment and efficacy, thus leading to them being more “likely to embed themselves and be enthusiastic about their work (engagement)” (p. 132). We therefore expect a solid moderate positive correlation between engagement and the CPC-12 in about a similar range as stated above.

### Gratitude

The close relationship of gratitude and PsyCap was demonstrated in a study by Luthans, Youssef [52], in which they discussed a range of possible constructs to be included into PsyCap. They regard gratitude as a promising aspirant for inclusion describing it as “the extra mile willingly traveled by those with high PsyCap” [52]. They find that gratitude prevents people from having negative labels and thoughts about their fellow men, thus decreasing the positivity in those relationships, which would in turn lead to a lower level of PsyCap. They state that being grateful helps us to maintain a positive outlook on life and positively reinforce each other. Maintaining a positive view on life resembles parts of optimism and hope.This is also reflected in other studies, which found positive relationships between gratitude and optimism [53–55], hope [54] and life satisfaction and happiness [55]. McCullough, Emmons [54] report moderate positive correlations between gratitude and optimism (*r* = .28−.58) as well as for gratitude and the two factors of hope (*r* = .18−.67). Considering the connection of gratitude to some of the PsyCap components and its actual consideration as a component itself, we expect a moderate positive correlation of gratitude with the CPC-12.

### Personality

A proactive personality is desirable at an individual level as it predicts life satisfaction [56], but also from an organization’s perspective as it is for example positively related to job performance [57]. It means for a person to have an enhanced ability and desire to control the surrounding environment in an active, self-determined way. These control tendencies facilitate effective coping with occupational stressors [56], thus showing similarities to resilience. Optimism also seems closely related to a proactive attitude. Schmitz and Schwarzer [58] identify optimistic expectancy as the quintessence of the construct and describe proactive people as considering life to be full of opportunities. They furthermore associate proactive attitude with Bandura’s self-efficacy, deeming the two to be very similar constructs [58]. We therefore expect a moderate to high positive correlation between proactive attitude and the CPC-12.

The “Big Five” personality traits extraversion (*r* = .36) and conscientiousness (*r* = .39) reportedly show the strongest relationship with PsyCap. The correlation of agreeableness with PsyCap (*r* = .06) is unremarkable, whereas openness (*r* = -.1) and neuroticism (*r* = -.12) show marginal negative correlations [19, 22]. The positive correlation with extraversion seems logical as it has also been found to be positively related to positive affect, life satisfaction and happiness [27].

Looking at some of the PsyCap components respectively, one study testing the relationship of resilience with personality traits found a strong positive correlation with extraversion (*r* = .61) and conscientiousness (*r* = .45) as well as a strong negative correlation with neuroticism (*r* = -.65). The correlation with agreeableness was unremarkable, but there was a small positive correlation with openness (*r* = .20) [59]. The strong negative relationship with neuroticism measures up to one’s expectations. Neurotic people are vulnerable to emotional distress and susceptible to negative emotions and poor coping [60]. Campbell-Sills, Cohan [59] explain the strong positive relationships to extraversion as likely reflecting “the benefits of positive affective style, capacity for interpersonal closeness, and high levels of social interaction and activity” (p. 594). They furthermore explicate that the positive relationship with resilience can be fully explained by the tendency of conscientious people to use task-oriented coping. Taking all these findings together we expect a similar pattern of correlations between the “Big Five” and the CPC-12, moderate positive correlations with extraversion and conscientiousness, a moderate negative correlation with neuroticism, a small correlation with openness and no correlation with agreeableness.

## Study 1—Methods

### Participants and Procedure

Study 1 consisted of a total of 334 participants. Thirteen people were excluded from the analysis (six due to implausible or missing job description e.g. “xxx”, seven due to implausible tenure). The remaining 321 participants averaged 34.89 years (SD = 12.78), 60% were women and 76.6% were employees, 8.4% were self-employed and 13.7% temporary workers. Participants worked on average 33.79 hours a week (SD = 13.39) and had been employed for half a month to 43 years (M_employment_ = 7.91 years, SD = 9.72). 48% of the participants were in possession of a university degree and another 25% graduated with the general qualification for university entrance. Participants were recruited by publishing the link to the survey in several online social media groups. The survey was conducted in German. All participants were volunteers, no compensation was supplied.

### Materials

All scales were surveyed using a 6-point response format ranging from 1 = “*strongly disagree*” to 6 = “*strongly agree”* in order to minimize potential effects of different response formats on the new scale.

#### Hope

Hope was evaluated using the six item short version of the State Hope Scale (SHS) [23]. The German version of this scale was derived by translating the original version of the State Hope Scale [23] into German (including a retranslation for verification purposes) according to the guidelines for cross-cultural adaptation proposed by Guillemin, Bombardier [61].

#### Optimism

Optimism was evaluated using two different scales. (a) The affective valence of the orientation towards the future-questionnaire (Affektive Valenz der Zukunftsorientierung, AFF) [62] with five items including one inverted item. Cronbach’s α of the AFF was .82. (b) The revised German version of the Life Orientation Test (LOT-R) [63] with ten items (four filler items and three items respectively tapping the subscales optimism and pessimism; [64].

#### Resilience

Resilience was assessed using the German thirteen item short version of the Resilience Scale (RS-13) [65].

#### Self-efficacy

Self-efficacy was evaluated using two different measures. (a) The German General Self Efficacy Scale (GSE) [66] with ten items. (b) The German ten item short version of the Occupational Self-Efficacy Scale (OSE) [67].

#### Psychological capital

PsyCap was surveyed using the German version of the Psychological Capital Questionnaire [19] with 24 items. Six items each measured the four subscales hope, optimism, resilience and self-efficacy.

#### Construction of Compound-Psychological-Capital-Scale

The five selected scales (SHS, AFF, LOT-R, RS-13, and GSE) constitute the pool of items from which the Compound-Psychological-Capital questionnaire (CPC-12) was developed. All four constructs (i.e., hope, resilience, optimism, and self-efficacy) should have equal weight; hence, the best three items of each construct in terms of content and face validity were taken into account. Furthermore, following the concept of rational construction [68], only those items were included which met our claim of universality and are therefore not solely relevant to the workplace. All scales for the item pool were included in this study in their full length to be able to control their correlations comparing PCQ and CPC-12.

### Data analysis

The fit of all tested structural equation models was examined using the criteria proposed by Hu and Bentler [69]. Beyond χ² significance testing these criteria comprise a standardized root-mean-square residual (SRMR) ≤ 0.08 in combination with at least one of the following fit indices: a root-mean-square error of approximation (RMSEA) ≤ 0.06, a lower bound of the 90% confidence interval of the RMSEA ≤ 0.06, a comparative fit index (CFI) ≥ 0.95, or a Tucker-Lewis-index (TLI) ≥ 0.95. The Satorra-Bentler adjusted χ² was calculated to adjust for non-normal distributions of the variables [70]. The confirmatory factor analyses were conducted using the “lavaan” package [71] of R statistical software [72]. Due to forced choice in the standardized questionnaires, there was no missing data.

## Study 1—Results

Table 1 presents descriptive statistics, Cronbach’s α and the correlation matrix for the study variables. The strong positive relationship (*r* = .70, *p* < .001) between the PCQ and the newly created CPC-12 alludes to the measurement of a similar but not identical construct. As hypothesized the correlation between CPC-12 and general self-efficacy is higher compared to the PCQ, while the correlation between CPC-12 and occupational self-efficacy is lower compared to the PCQ.

**Table 1:** Descriptive statistics and inter-correlations for study 1.

	*M*	*SD*	Hope	Optimism (AFF)	Optimism (LOT-R)	Resilience	General Self-Efficacy	Occupational Self-Efficacy	PsyCap (PCQ)	PsyCap (CPC-12)
Hope	4.25	1.04	.*84*							
Optimism (AFF)	4.83	0.99	.49***	.*82*						
Optimism (LOT-R)	4.41	1.04	.44***	.58***	.*74*					
Resilience	4.64	0.91	.56***	.46***	.41***	.*79*				
General Self-Efficacy	4.22	0.86	.55***	.42***	.35***	.70***	.*88*			
Occupational Self-Efficacy	4.29	0.93	.56***	.47***	.39***	.56***	.67***	.*85*		
PsyCap (PCQ)	4.51	1.03	.61***	.56***	.55***	.59***	.55***	.77***	.*92*	
PsyCap (CPC-12)	4.44	0.99	.79***	.65***	.53***	.80***	.82***	.71***	.70***	.*82*

Notes: Cronbach’s α is displayed diagonal; AFF = Affektive Valenz der Zukunftsorientierung (Affective valence of the future orientation), LOT-R = Life Orientation Test—Revised, PsyCap = Psychological Capital, PCQ = Psychological Capital Questionnaire, CPC-12 = Compound Psychological Capital Scale.; p-scores:

* <.05,

** <.01,

*** <.001.

Table 2 exhibits measurement models for all selected scales, including the PCQ measure for PsyCap. To examine the expected factorial structure of the PCQ, we conducted a confirmatory factor analysis (CFA). We began by fitting this model with the six items for each facet (i.e., hope, resilience, optimism, and self-efficacy) and then fit each of the four dimensions to the higher-order PsyCap. The estimates of model fit (SRMR = .062, RMSEA = .061, CFI = .841) are not acceptable according to Hu and Bentler [69].

**Table 2:** Measurement models for study 1 using MLM estimator.

	N factors	X²	df	p	SRMR	TLI	CFI	RMSEA	RMSEA 90%-CI
PsyCap (PCQ)	4+g	549.04	248	<.001	.062	.823	.841	.061	.055−.068
PsyCap (CPC-12)	4+g	77.727	50	.007	.046	.950	.962	.042	.024−.058

Notes: PsyCap = Psychological Capital, PCQ = Psychological Capital Questionnaire, CPC-12 = Compound Psychological Capital Scale.

To confirm the expected higher-order factor of PsyCap in the CPC-12, we conducted a CFA on the data analogous to the one above. We began by fitting this model with three items for each facet (i.e., hope, resilience, optimism, and self-efficacy) and then fit each of the four dimensions to the higher-order PsyCap. Results indicated the following estimates of model fit: SRMR = .046, RMSEA = .042, CFI = .962. The model seems to be a very good fit with all three indices meeting the cutoff criteria by Hu and Bentler [69].

**Fig 1:**
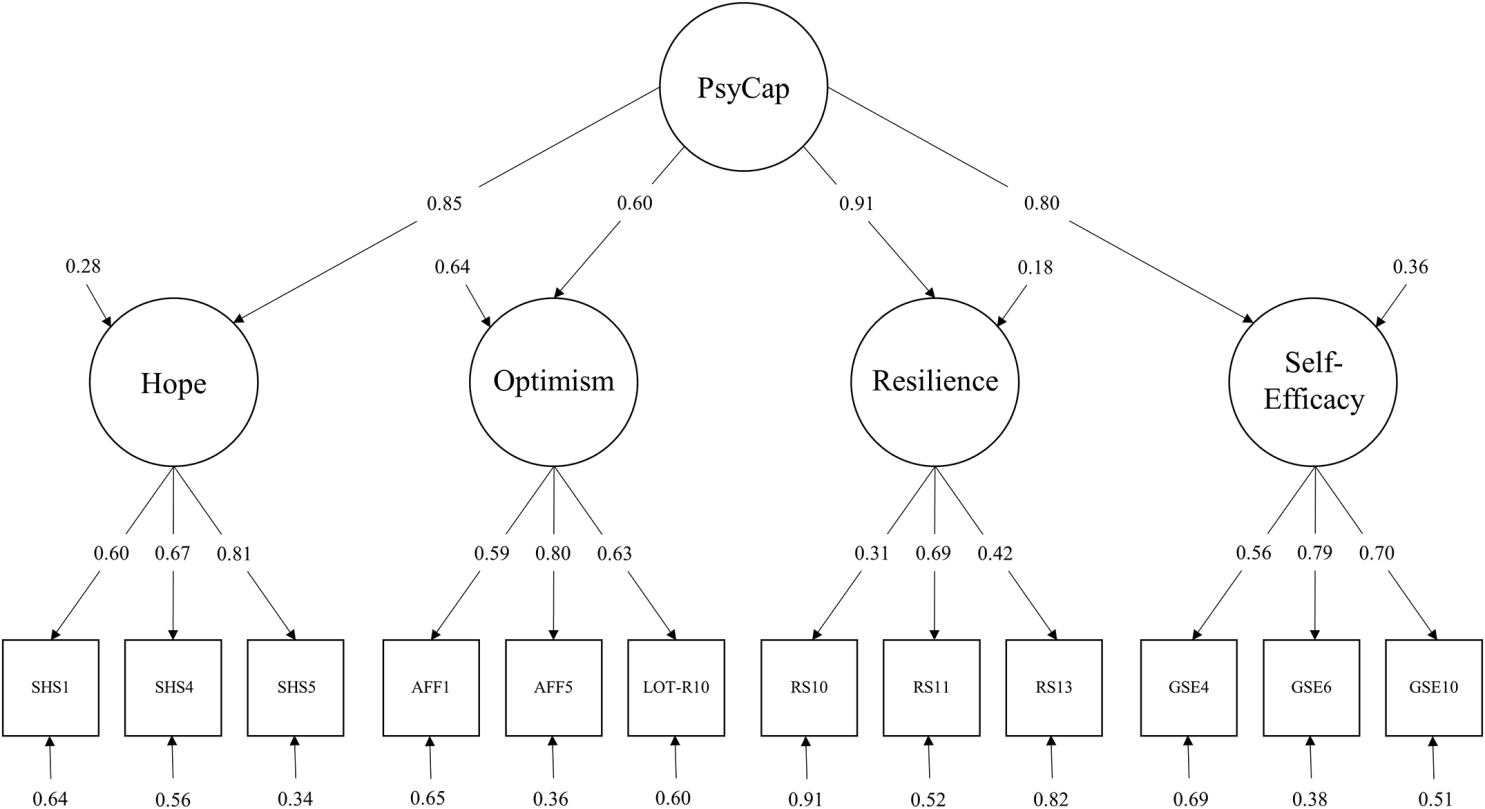
Four (plus g-) factor measurement model for Psychological Capital. Abbreviated items refer to S1 Appendix.

Furthermore, each of the factor loadings was significant on their respective latent factor at *p <* .01 (Fig 1). The confirmatory factor analysis in Study 1 supports the proposed higher-order factor structure for the newly created PsyCap measure CPC-12.

## Study 2—Methods

### Participants and Procedure

Sample 2 consisted of a total of 202 participants (82.7% employees, 9.4% self-employed, and 7.9% temporary workers) between 18–72 years (M_age_ = 37.79, SD = 13.10). 72.3% were female. Participants worked on average 35.74 hours a week (SD = 11.18) and had been employed for one month to 45 years (M_employment_ = 9.37 years, SD = 9.74). 35.1% were in possession of a university degree and another 25.2% graduated with the general qualification for university entrance. Participants were recruited by publishing the link to the survey in several social media groups. The survey was conducted in German. All participants were volunteers, no compensation was supplied.

### Materials

#### Psychological capital

PsyCap was measured with the CPC-12 (Study 1), using a 6-point response format ranging from 1 = “*strongly disagree*” to 6 = “*strongly agree”*.

#### Positive and negative affect

PA and NA were evaluated using the Positive and Negative Affect Schedule [73]. Participants responded with 20 items to the question asking how they felt “during the past two weeks” (1 = *“very slightly or not at all”* to 5 = *“very much”)*. Ten items measured positive affect (e.g. excited, attentive) and ten items measured negative affect (e.g. guilty, afraid). The items were arranged randomly.

#### Job satisfaction

Job satisfaction was measured using three items [35, 74]. The first item measured general job satisfaction (“All things considered are you satisfied with your job?”), which participants were able to answer with “yes” or “no”. The second item (“How satisfied are you with your job in general?”) was rated using a 5-point scale from 1 = *“very dissatisfied”* to 5 = *“very satisfied”*. The third item asked participants to rate the percentage of time they feel satisfied, unsatisfied or neutral with their job in general (e.g. “The percent of time I feel satisfied with my present job.”). The analysis was conducted using the mean-score of the z-standardized items.

#### Satisfaction with life

Life satisfaction was measured using the Satisfaction with Life Scale [75], using a 5-point response format ranging from 1 = *“strongly agree”* to 5 = *“strongly disagree”*. Participants rated five given statements (e.g. “I am satisfied with my life.”).

#### Subjective well-being

The standardized z-scores of the four previously described scales were averaged to create subjective well-being. PA, NA and satisfaction with life were included because Arthaud-Day, Rode [76] found the three-factor model of subjective well-being consisting of PA, NA and life satisfaction to be superior to any other two- or one-factor model and the best fit to their data. Job satisfaction was then included to add a domain-specific focal point on work [35, 36] for the comparability to previous studies on well-being and PsyCap.

#### Perceived social support

A short version of the Perceived Support Questionnaire [77] was used to measure perceived social support. Using a 5-point response format ranging from 1 = *“strongly disagree”* to 5 = *“strongly agree”* participants had the possibility to rate to what extent the six given statements (e.g. “There is someone very close to me whose help I can always count on.”) fit their own lives.

#### Meaning of work

The Work and Meaning Inventory [78] was used to measure meaning of work. Using a 5-point response format ranging from 1 = *“absolutely untrue”* to 5 = *“absolutely true”* participants rated to what extent the ten given statements (e.g. “My work helps me make sense of the world around me.”) applied to them.

#### Engagement

To measure engagement the Utrecht Work Engagement Scale [79] was used. Given a 7-point Likert scale ranging from 1 = *“never”* to 7 = *“always”* participants were asked to rate nine presented statements (e.g. “I am immersed in my job.”).

#### Gratitude

Gratitude was evaluated using the Gratitude-Questionnaire [54]. Given a 7-point Likert scale ranging from 1 = *“strongly disagree”* to 7 = *“strongly agree”* participants were asked to rate to what extent the six presented statements (e.g. “I have so much in life to be thankful for.”) applied to them.

#### Proactive attitude

The Proactive Attitude Scale [58] was used to measure proactive attitude. Using a 4-point response format ranging from 1 = *“not at all true”* to 4 = *“exactly true”* participants rated to what extent the eight given statements (e.g. “I can choose my own actions.”) applied to them.

#### “Big Five”

The five personality traits extraversion, neuroticism, conscientiousness, agreeableness and openness were assessed using the Big Five Inventory (BFI-S) [80]. Participants rated 15 statements (e.g. “I see myself as someone who is outgoing, sociable.”) on a 7-point Likert scale from 1 = *“does not apply to me at all”* to 7 *= “applies to me perfectly”*.

### Data Analysis

The fit of all tested structural equation models was examined using the same criteria as presented in study 1 [69]. According to these indices the model for subjective well-being, which consisted of four independent constructs, showed an acceptable fit when tested for the unidimensional character of the compound variable using CFA (Satorra-Bentler-χ ² (2, 202) = 4.172, *p* <.125, CFI = .957, SRMR = .035, RMSEA = .073, CI_RMSEA_ = .00−.15).

The data analysis was run using the statistical software R [72]. The confirmatory factor analyses were conducted using the “lavaan” package [71], other used packages were “Hmisc” and “pastecs” [81].

We used multiple imputation methods [82] to impute the three missing item responses prior to the statistical analysis. This maximizes power [83] and produces accurate parameter estimates [84].

## Study 2—Results

Results of the CFA for the CPC-12 indicated the following estimates of model fit: Satorra-Bentler-χ ² (50, 202) = 72.32, *p* <.021, CFI = .955, SRMR = .052, RMSEA = .047, CI_RMSEA_ = .022−.068. All the indices can be deemed to be a good model fit according to Hu and Bentler [69].

Table 3 presents descriptive statistics, Cronbach’s α and bivariate correlations for the variables of study 2. All correlations are according to our hypotheses. Subjective well-being (*r* = .58), proactive attitude (*r* = .57) and positive affect (*r* = .54) showed the highest positive correlations, agreeableness showed no substantive correlation with the CPC-12, neuroticism and negative affect showed negative correlations.

**Table 3:** Descriptive statistics and inter-correlations for study 2.

	*M*	*SD*	PsyCap	SWB^a^	PA	NA^b^	JS^a^	LS	PSS	MoW	Eng	Grat	ProA	Con	Extr	Neur	Open	Agree
PsyCap	4.54	0.50	.*81*															
SWB^a^	0^a^	0.68^a^	.58***	*-*														
PA	3.40	0.60	.54***	.64***	.*86*													
NA	4.22	0.62	-.25***	-.68***	-.16*	.*64*												
JS^a^	0^a^	0.81^a^	.40***	.70***	.30***	-.33***	.*72*											
LS	3.74	0.63	.39***	.71***	.29***	-.38***	.27***	.*74*										
PSS	4.42	1.3	.22**	.41***	.14	-.35***	.18*	.47***	.*87*									
MoW	3.64	0.76	.28***	.50***	.38***	-.25***	.43***	.30***	.16*	.*91*								
Eng	4.77	0.65	.39***	.60***	.51***	-.24***	.52***	.38***	.21**	.70***	.*95*							
Grat	5.88	0.8	.27***	.43***	.26***	-.23***	.22**	.46***	.45***	.30***	.33***	.*68*						
ProA	3.07	0.39	.57***	.54***	.39***	-.35***	.30***	.46***	.48***	.41***	.48***	.48***	.*67*					
Con	5.48	0.89	.29***	.34***	.29***	-.27***	.17*	.19**	.17*	.24***	.37***	.16*	.35***	.*55*				
Extr	5.12	1.11	.24***	.22**	.18*	-.20*	.05	.17*	.30***	.22**	.31***	.25***	.34***	.18*	.*70*			
Neur	3.96	1.21	-.49***	-.35***	-.20**	.34***	-.17*	-.24***	-.20**	-.17*	-.23***	*-*.16*	*-*.37***	*-*.*12*	*-*.19**	.*70*		
Open	5.19	1.04	.20**	.21**	.24***	-.13	.10	.10	.18**	.32***	.33***	.25***	.30***	.19**	.37***	.*-08*	.*59*	
Agree	5.34	0.88	.04	.14*	.02	-.13	.07	.18**	.23**	.17*	.17*	.24***	.19**	.18*	.*02*	.*06*	.*11*	.*37*

Notes: Cronbach’s αs are displayed diagonal; CPC-12 = Compound Psychological Capital Scale, SWB^a^ = aubjective well-being, PA = positive affect, NA = negative affect, JS^a^ = job satisfaction, LS = satisfaction with life, PSS = perceived social support, MoW = meaning of work, Eng = engagement, Grat = gratitude, ProA = proactive attitude, Con = conscientiousness, Extr = extraversion, Neur = meuroticism, Open = openness, Agree = agreeableness; p-scores:

* <.05,

** <.01,

*** <.001.

^a^ standardized z-scores

## Discussion

Since its emergence in 2004, the construct of PsyCap is assessed using the PCQ as the standard measure in more than 14 countries and languages, along with slight alterations of the scale to match the target group’s needs [85]. The domain-specific measure itself is tied to the working world, although studies indicate associations with psychological constructs important for students, the unemployed, and retirees alike [20]. Therefore, the aim of this study was to develop and validate a German compound measure for PsyCap, with the general claim of being applicable to all domains of life.

According to our results, the CPC-12 fits the proposed model of PsyCap very well. The four subscales hope, optimism, resilience, and self-efficacy are identifiable as subcomponents of the overall measure while the higher-order factor can incrementally explain additional variance in the data. The moderate to high correlations to other work-related (MOW, job satisfaction and engagement; r = .28−.40) and more general constructs of positive psychology (i.e. subjective well-being, proactive attitude, and gratitude; r = .22.—.58) are comparable to previous research on PsyCap and speak for the external validity of the CPC-12. The results indicate that PsyCap could in fact be a general construct and applicable to more areas than POB. By abstaining from items with work related connotations, the CPC-12 can be applied to many fields of interest, i.e. sports and education. The CPC-12 is also an alternative in work-related research for areas where the item wordings of the PCQ might not be suitable, i.e. volunteering or small organizations, since its correlations with work-related constructs are similar to the PCQ. It is important to notice that while the CPC-12 is a short and economic way to measure PsyCap, we recommend using the original scales we used for the item pool if a specific sub-facet is the key area of interest.

## Future Directions

To achieve these desirable outcomes, future research should address the implementation of interventions that increase PsyCap. Although PsyCap is open to development and has already been proven to be alterable through interventions [5, 86], organizations to date still fail to increase their efforts to enhance this underemphasized positive core construct. We go one step further and propose to not only implement PsyCap interventions at the work place, but at schools, universities, or even in families. These interventions can be carried out on a group-level or as tailored training interventions based on individual scores. With the CPC-12 we provide a reliable tool to measure those individual PsyCap scores in many domains of life. In order to facilitate a reasonable interpretation of the scores, the quality criterion of standardization of the CPC-12 will have to be addressed to create up-to-date norms and to define the population to which they apply. The generalizability of the CPC-12 should be re-evaluated using different samples in other life-domains.

All in all, PsyCap seems to be multifarious and connected to a wide variety of other positive psychological constructs. High correlations with well-being, life satisfaction or job satisfaction make a case for PsyCap to become a focus of prospective research in positive psychology. We believe that people high in PsyCap are more likely to lead flourishing lives and probably even more likely to build a flourishing society.

## Limitations

The following limitations should be kept in mind when interpreting the results. The high correlations between the CPC-12 and the scales used to create the CPC-12 in study 1 are in part due to common items in these scales and should not be interpreted without this context.

All participants were recruited online, which entails that findings may not generalize to people not using the internet or social networks. Although Gosling, Vazire, Srivastava and John [87] agree that data from the internet is not free from methodological constraints, they do emphasize that samples using online recruitment are as diverse, adjusted, at least as good in quality as most traditional methods and “not as flawed as is commonly believed” (p. 102). The use of a nonprobability sample in this study raises further concerns about generalizability.

In addition, the scales for gratitude (α = .68) and proactive attitude (α = .67) lack reliability (smaller than .70). Results should therefore be considered with caution. The same caution should apply to the five personality traits as they also lack reliability. We found low merits for conscientiousness (α = .55), openness (α = .59) and agreeableness (α = .37), the latter being extremely low.

## Ethics Statement

This study is in accordance with the APA ethical principles regarding research with human participants. This study does not involve any conflict of ethics, since no clinical intervention was performed. Neither were blood or tissue samples taken for study purposes.

Participants were informed before participating that their responses would be treated confidentially and anonymously and that all data would be analyzed in a generalized manner so that no conclusions could be drawn about individual persons. The participants were informed that they would give their consent by proceeding past the welcome page of the online survey. This procedure is in accordance with the Freie Universität Berlin ethics committee’s guidelines. There was no contact between researchers and participants. Participation in this study was voluntary. This study was approved by the ethics committee of Freie Universität Berlin ID 102/2015.

## Supporting Information

S1 Appendix Interview questionnaire on barriers to employment and their overcoming. (PDF)

S1 Dataset Dataset study 1. (CSV)

S2 Dataset Dataset study 2. (CSV)
