# Supplementary material for: Measuring Psychological Capital: Construction and Validation of the Compound PsyCap Scale (CPC-12)
Source: PLoS One. 2016 Apr 1;11(4):e0152892. doi: 10.1371/journal.pone.0152892 (PMC4817957; doi:10.1371/journal.pone.0152892)
Supplement: S1 Appendix — (PDF) [file pone.0152892.s001.pdf]

S1 Appendix

CPC-12 Scale (German)

1. Sollte ich mich in einer Zwickmühle befinden, würden mir viele Auswege einfallen. (SHS1)[a]

2. Im Moment betrachte ich mich als recht erfolgreich. (SHS4)[a]

3. Mir fallen viele Strategien ein, um meine derzeitigen Ziele zu erreichen. (SHS5)[a]

4. Ich freue mich auf das Leben, das noch vor mir liegt. (AFF1)[b]

5. Die Zukunft wird für mich viel Gutes mit sich bringen. (AFF5)[b]

6. Alles in allem erwarte ich, dass mir mehr gute als schlechte Dinge widerfahren. (LOT-R10)[c]

7. Ich kann mich auch überwinden, Dinge zu tun, die ich eigentlich nicht machen will. (RS10)[d]

8. Wenn ich in einer schwierigen Situation bin, finde ich gewöhnlich einen Weg heraus. (RS11)[d]

9. Ich kann es akzeptieren, wenn mich nicht alle Leute mögen. (RS13)[d]

10. In unerwarteten Situationen weiß ich immer, wie ich mich verhalten soll. (GSE4)[e]

11. Wenn ein Problem auftaucht, kann ich es aus eigener Kraft meistern. (GSE10)[e]

12. Schwierigkeiten sehe ich gelassen entgegen, weil ich mich immer auf meine Fähigkeiten verlassen kann. (GSE6)[e]

CPC-12 Scale (English)

1. If I should find myself in a jam, I could think of many ways to get out of it.[a]

2. Right now, I see myself as being pretty successful.[a]

3. I can think of many ways to reach my current goals.[a]

4. I am looking forward to the life ahead of me.[b]

5. The future holds a lot of good in store for me.[b]

6. Overall, I expect more good things to happen to me than bad.[c]

7. Sometimes I make myself do things whether I want to or not.[d]

8. When I'm in a difficult situation, I can usually find my way out of it.[d]

9. It's okay if there are people who don't like me.[d]

10. I am confident that I could deal efficiently with unexpected events.[e]

11. I can solve most problems if I invest the necessary effort.[e]

12. I can remain calm when facing difficulties because I can rely on my coping abilities.[e]

Notes: [a] State Hope Scale (SHS), [b] Affective Valence of the Orientation towards the Future-Questionnaire (AFF), [c] Life-Orientation-Test (LOT-R), [d] Resilience Scale (RS-13), [e] The General Self-Efficacy Scale (GSE)
